# Co-expression of wild-type FLT3 attenuates the inhibitory effect of FLT3 inhibitor on FLT3 mutated leukemia cells

**DOI:** 10.18632/oncotarget.10147

**Published:** 2016-06-17

**Authors:** Fangli Chen, Yuichi Ishikawa, Akimi Akashi, Tomoki Naoe, Hitoshi Kiyoi

**Affiliations:** ^1^ Department of Hematology and Oncology, Nagoya University Graduate School of Medicine, Nagoya, Japan; ^2^ Department of Hematology/Oncology Research, National Hospital Organization, Nagoya Medical Center, Nagoya, Japan

**Keywords:** AML, FLT3 inhibitor, resistance, Wt-FLT3, FLT3 ligand

## Abstract

*FLT3* mutation is found in about 30% of acute myeloid leukemia (AML) patients and is associated with a poor prognosis. Several FLT3 inhibitors are undergoing investigation, while their clinical efficacies were lower than expected and several resistant mechanisms to FLT3 inhibitors have been demonstrated. Although most AML cells harboring *FLT3* mutation co-express wild-type (Wt)-FLT3, it is not fully understood how Wt-FLT3 expression is associated with the resistance to FLT3 inhibitors. In this study, we elucidated a resistant mechanism by which FL-dependent Wt-FLT3 activation reduced inhibitory effects of FLT3 inhibitors. We demonstrated that FL-stimulation much more strongly reduced growth inhibitory effects of FLT3 inhibitors on Wt- and mutant-FLT3 co-expressing cells than sole mutant-FLT3 expressing cells both in vitro and in vivo. It was also confirmed that FL impaired the anti-leukemia effects of FLT3 inhibitors on primary AML cells. We elucidated that FL impeded the inhibitory effects of FLT3 inhibitors mainly through the activation of Wt-FLT3, but not mutated FLT3, in the Wt- and ITD-FLT3 co-expressing cells. Furthermore, FL-induced activation of Wt-FLT3-MAPK axis was the dominant pathway for the resistance, and the glycosylation of Wt-FLT3 was also vital for FL-dependent kinase activation and following resistance to FLT3 inhibitors. Thus, we clarified the importance of co-expressing Wt-FLT3 in resistance to FLT3 inhibitors. These findings provide us with important implications for clinical application and new strategies to improve clinical outcomes of FLT3 inhibitors.

## INTRODUCTION

The mutations in FMS-like tyrosine kinase 3 (*FLT3*) gene are frequently identified in acute myeloid leukemia (AML) and consist of internal tandem duplication (ITD) in the juxtamembrane domain and point mutation or deletion in the tyrosine kinase domain (KDM) [1–3]. *FLT3*-ITD mutation has been reported in 20-30% of patients with AML and is associated with an increased risk of relapse and poor prognosis [3, 4]. FLT3 is expressed on normal hematopoietic stem and progenitor cells and plays important roles in cell proliferation, anti-apoptosis and survival in normal hematopoiesis [5]. The binding of FLT3 ligand (FL) to FLT3 receptor initiates its dimerization and activates downstream molecules; however *FLT3*-ITD mutation constitutively activates downstream molecules and induces autonomous cell proliferation to cytokine dependent cell lines *in vitro* [6–9].

Because of its clinical and biological significance, mutated FLT3 is an attractive therapeutic target to cure AML patients. Over the last decade, many agents have been investigated as FLT3 inhibitors and evaluated for their efficacy and safety in clinical trials. The first generation FLT3 inhibitors, including sunitinib, lestaurtinib and midostaurin have been evaluated either as monotherapy or in combination with cytotoxic chemotherapy. However, patients demonstrated only a transient response in monotherapy [10–13]. Due to the dismal clinical efficacy of first generation FLT3 inhibitors, second generation inhibitors were screened for sensitivity and selectivity against mutant FLT3 kinase. Quizartinib demonstrated enhanced potency and selectivity for FLT3 *in vitro* and a significantly longer half-life for sustained FLT3 inhibition *in vivo* [14].

Despite promising outcomes in preclinical models, the clinical efficacy of FLT3 inhibitors was lower than expected and no FLT3 inhibitors have been approved to date. Several resistance mechanisms, such as potency, plasma protein binding and emergence of secondary mutation in the kinase domain have been revealed [15–17]. Recently, it was reported that increased plasma FL concentration after chemotherapy impeded the efficacies of FLT3 inhibitors [18]. However, the mechanism of reduced inhibitory effects of FLT3 inhibitors caused by FL has not been fully elucidated. Since most AML cells harboring *FLT3* mutation co-express wild type (Wt)-FLT3, we hypothesized that FL stimulation to the Wt-FLT3 and its downstream molecules mainly conferred resistance to FLT3 inhibitors in FLT3 mutated AML cells. In this study, we established Wt- and ITD-FLT3 co-expressing cells and performed a systematic comparison of five FLT3 inhibitors. Then, the impact of FL stimulation on the efficiency of those inhibitors was investigated in established co-expressing cells, as well as primary patient AML samples *in vitro and in vivo*. Our results elucidated a novel resistance mechanism that FL attenuated inhibitory effects of FLT3 inhibitors mainly through the activation of Wt-FLT3, not mutated FLT3. This finding also has important implications for clinical application and screening systems for new FLT3 inhibitors. Moreover, we indicated the strategies to overcome this resistance mechanism.

## RESULTS

### FL reduces growth inhibitory effects of FLT3 inhibitors on Wt-FLT3 co-expressing cells

**Figure 1:**
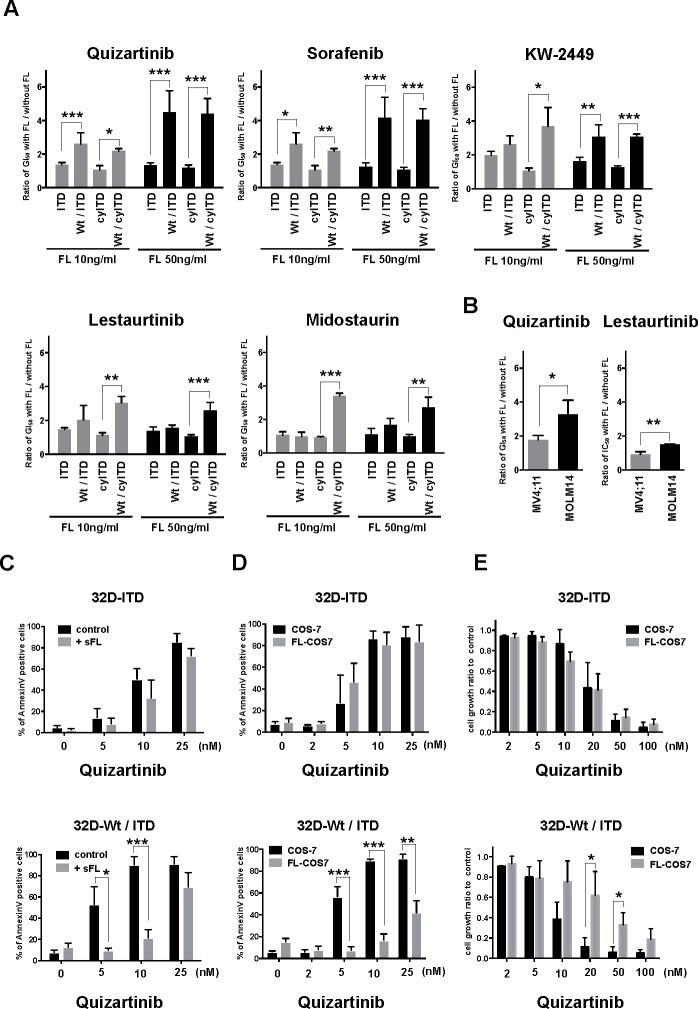
FL impairs effects of FLT3 inhibitors on Wt-FLT3 co-expressing cells **A.** Mutant-FLT3 32D cells were treated with increasing concentrations of the indicated FLT3 inhibitors in the presence or absence of FL at 10 or 50ng/ml for 48 hours. Fold changes to GI_50_ values without FL are shown. **B.** MV4;11 cells and MOLM14 cells were treated with increasing concentrations of quizartinib or lestaurtinib with or without 50ng/ml FL for 72 hours. Fold changes to GI_50_ values without FL are shown. **C.** Sole ITD-FLT3 expressing 32D cells and Wt- and ITD-FLT3 co-expressing cells were treated with increasing concentrations of quizartinib with or without FL at 50ng/ml for 24 hours and then processed to measure the binding of Annexin V and PI by flow cytometry to assess induction of apoptosis. **D.** 32D cells were co-cultured with COS-7 or FL-COS-7 cells overnight, followed by exposure to varying concentrations of quizartinib for 48 hours to assess apoptosis induction. **E.** MTT assay was performed at 24 hours after exposure to varying concentrations of quizartinib in 32D and COS-7 or FL-COS-7 co-culture condition. Error bars represent the mean values ± S.D. from at least three independent experiments (*P < 0.05, **P < 0.01, ***P < 0.001).

We evaluated growth inhibitory effects of five FLT3 inhibitors, quizartinib, sorafenib, KW-2449, midostaurin and lestaurtinib in the presence or absence of exogenous FL using sole ITD-FLT3 expressing 32D cells, and Wt- and ITD-FLT3 co-expressing 32D cells (Figure 1A and Supplementary Figure 1). Compared with sole ITD-FLT3 cells, FL stimulation significantly increased GI_50_ values in Wt- and ITD-FLT3 co-expressing cells dose dependently. The GI_50_ value of quizartinib against sole ITD-FLT3 expressing cells was 4.94 nM compared with 6.56 nM in the presence of 50 ng/mL exogenous FL. Although FL stimulation weakly impaired the inhibitory effect of quizartinib against sole ITD-FLT3 cells, the GI_50_ value was significantly increased 4.5-fold with FL stimulation in Wt- and ITD-FLT3 co-expressing cells (GI_50_ = 4.21 nM and 19.1 nM, *P* = 0.011). A similar pattern of elevated GI_50_ in co-expressing 32D cells was observed with sorafenib and, at a low extent, KW-2449 (*P* = 0.01 and 0.001, respectively). However, the inhibitory effects of midostaurin and lestaurtinib against Wt- and ITD-FLT3 co-expressing cells were not impaired by the FL stimulation. To confirm these results excluding the effect of FL on ITD-FLT3, we also evaluated the inhibitory effects of these inhibitors on sole cytoplasmic (cy-) ITD-FLT3, which lacked an extracellular domain of ITD-FLT3, expressing cells and Wt- and cyITD-FLT3 co-expressing cells (Figure 1A and Supplementary Figure 1). Due to the lack of the extracellular domain, the GI_50_ values of these inhibitors against cyITD-FLT3 expressing cells were not affected by FL. However, when Wt-FLT3 co-expressed in cyITD-FLT3 cells, the GI_50_ values were significantly elevated by exogenous FL (quizartinib 4.2-fold, *P* < 0.0001; sorafenib 3.86-fold, *P* = 0.003; KW-2449 3.1-fold, *P* < 0.0001; lestaurtinib 2.6-fold, *P* < 0.0001; midostaurin 2.62-fold, *P* = 0.03) (Figure 1A). Quizartinib and sorafenib has higher potency against ITD-FLT3 than Wt-FLT3, as GI_50_ values against Wt-FLT3 were much higher than against ITD-FLT3 (3.68-fold, *P* = 0.02 and 2.55-fold, *P* = 0.02, respectively). Midostaurin and lestaurtinib showed similar potencies against both ITD-FLT3 and Wt-FLT3 (Supplementary Figure 2). These results indicate that different potencies against ITD-FLT3 and Wt-FLT3 are associated with the reduction of growth inhibitory effects on Wt- and ITD-FLT3 co-expressing cells. On the other hand, midostaurin and lestaurtinib showed significantly higher potency against cyITD-FLT3 than Wt-FLT3 (*P* = 0.03 and *P* = 0.002, respectively), resulting in the high GI_50_ ratio of Wt- and cyITD-FLT3 co-expressing cells to sole cyITD-FLT3 cells.

We confirmed that co-expression of Wt-FLT3 attenuated the growth inhibitory effects of FLT3 inhibitors on ITD-FLT3 expressing 32D cells by another 3 independent clones (Data not shown). Furthermore, we also confirmed these data in Wt- and ITD-FLT3 co-expressing FDC-P1 cells (Data not shown). Consistent with these results, the GI_50_ value of quizartinib against MOLM-14, a human leukemia cell line expressing both Wt- and ITD-FLT3, was significantly increased by FL compared with MV4;11 cells harboring a homozygous ITD allele, while the impaired inhibitory effect of lestaurtinib caused by FL was limited in MOLM-14 cells (Figure 1B).

Addition of soluble FL significantly decreased the quizartinib-induced apoptosis in Wt- and ITD-FLT3 co-expressing 32D cells, but not in sole ITD-FLT3 cells (Figure 1C). FL transcripts have two isoforms and are translated into soluble FL and membrane-bound form FL, respectively. The membrane-bound form FL is expressed on stromal cells in the bone marrow microenvironment and it is proposed that a bone marrow niche protects leukemia cells from chemotherapy and TKIs treatment [19, 20]. We therefore established membrane-bound form FL-expressing COS-7 cells to replicate the bone marrow microenvironment consisting of FL-expressing stromal cells (Supplementary Figure 3A-3B). Then, we evaluated the inhibitory effect of quizartinib on sole ITD-FLT3 cells and Wt- and ITD-FLT3 co-expressing cells under co-culture conditions with COS-7 or FL-COS-7. In co-culture with FL-COS-7 cells, the inhibitory effect of quizartinib on Wt- and ITD-FLT3 co-expressing cells was significantly decreased, but not on sole ITD-FLT3 cells in both MTT assay and apoptosis assay (Figure 1D-1E). These results indicated that FL-stimulation reduced the growth inhibitory effects of FLT3 inhibitors on Wt- and ITD-FLT3 co-expressing cells both in liquid and cell-contact conditions.

### FL reduces anti-leukemia effect of FLT3 inhibitors on Wt-FLT3 co-expressing cells *in vivo*

**Figure 2:**
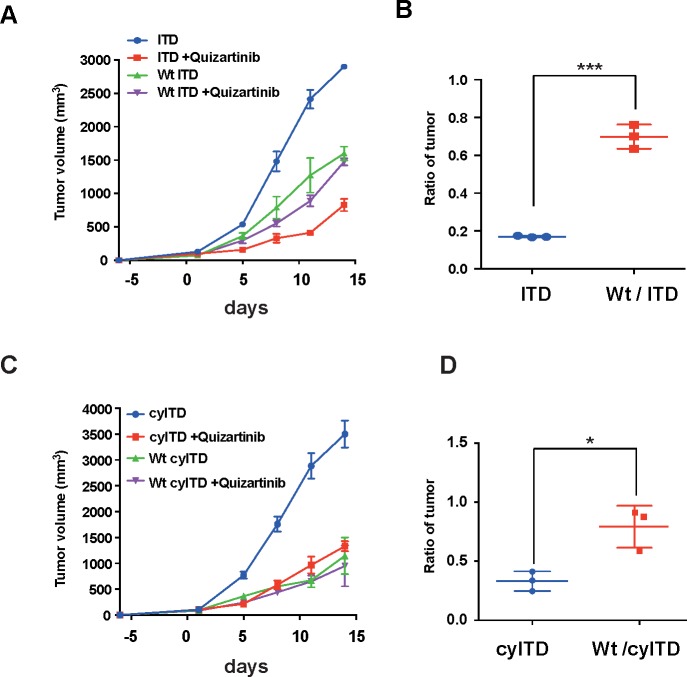
FL attenuate anti-leukemia effect of FLT3 inhibitors *in vivo* **A.** 1×10^7^ FLT3-ITD expressing cells or Wt- and ITD-FLT3 co-expressing cells were inoculated subcutaneously into a shaved area of six NOD-SCID mice for each cell line. At seven days after inoculations, six mice from each cell line were divided into vehicle and quizartinib treatment groups. Quizartinib (1mg/kg q.d.) was orally administrated for 14 days. The graph shows the average volume of tumors corresponding to each cell line. **B.** The relative ratio of quizartinib-treated tumor volume to control tumor volume on day 11 is represented. Inhibitory effects of FLT3 inhibitors on co-expressing cells were much more reduced by the presence of FL in plasma than sole ITD-FLT3 expressing cells. **C**. and **D**. FL also impaired *in vivo* effects of quizartinib on Wt- and cyITD-FLT3 co-expressing cells. Error bars represent the mean values ± S.D. from at least three independent experiments (*P < 0.05, **P < 0.01, ***P < 0.001).

We next examined whether FL reduced the inhibitory effects of FLT3 inhibitors *in vivo.* We inoculated subcutaneously sole ITD-FLT3 cells and Wt- and ITD-FLT3 co-expressing cells into NOD/SCID mice, and treated them with 1 mg/kg of quizartinib or vehicle control. In this experiment, we inoculated human FLT3 expressing 32D cells into NOD/SCID mice; however we confirmed that that mouse FL activates human FLT3 in consistent with previous report [21]. In this model, oral administration of quizartinib showed a potent anti-tumor effect on sole ITD-FLT3 cell-inoculated mice, while the inhibitory effect of quizartinib was significantly lower in Wt- and ITD co-expressing cell-inoculated mice than sole ITD-FLT3 cell-inoculated mice (Figure 2A-2B). We obtained the same results from the experiment using Wt- and cyITD-FLT3 expressing cells (Figure 2C-2D).

### FL activates only Wt-FLT3 in Wt-FLT3 co-expressing cells and reduces the kinase inhibitory effect of FLT3 inhibitors

**Figure 3:**
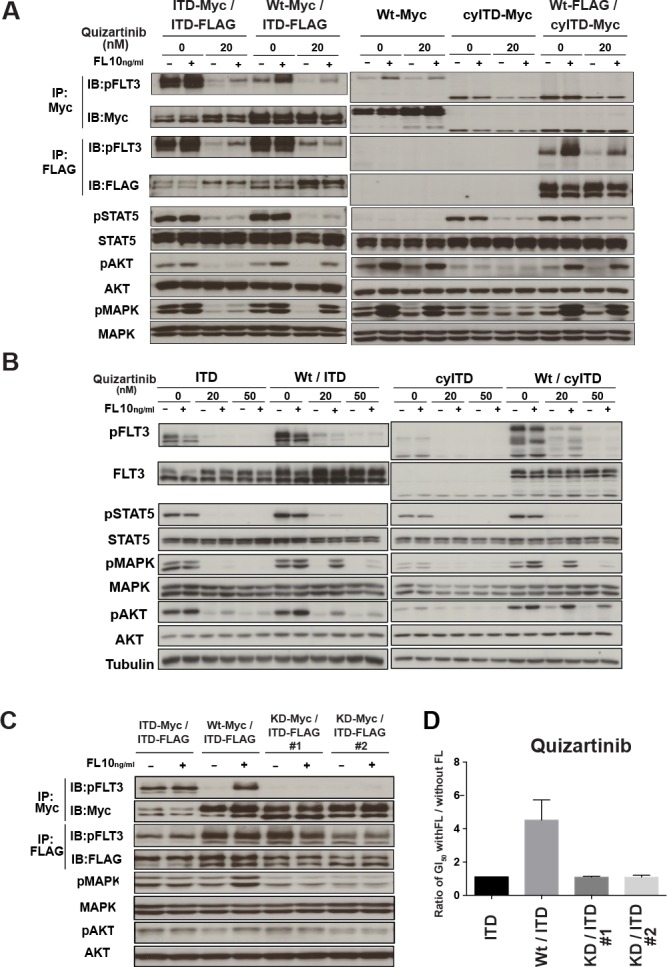
FL-dependent Wt-FLT3 signals reduced the inhibitory effect of FLT3 inhibitors **A.** The established 32D cells were treated with quizartinib for 2h, followed by 10ng/ml FL stimulation for 10 minutes. Wt-FLT3 and mutant FLT3 were immunoprecipitated by each tag as indicated and subjected to Western blot. Phosphorylation levels of FLT3, STAT5, AKT and MAPK were examined. **B.** 32D cells were treated with quizartinib at the indicated concentrations in the presence or absence of 10ng/ml FL for 2 hours. Phosphorylation levels of FLT3, STAT5, AKT and MAPK were detected by Western blot. **C.** Established kinase dead-FLT3 and ITD-FLT3 co-expressing 32D cells were stimulated with FL and phosphorylation levels of FLT3, AKT and MAPK was determined by Western Blot. **D.** kinase dead FLT3 co-expressing ITD-FLT3 cells were treated by quizartinib with of without FL. Fold changes to GI_50_ values without FL are shown. All results are representative of at least three independent experiments.

We next evaluated the effects of FL on FLT3 and its downstream molecules. Cells were treated with quizartinib for 2 hours and followed by FL stimulation for 10 minutes. This model simulated the *in vivo* condition as soluble FL was secreted by bone marrow stromal cells continuously. In sole ITD-FLT3 cells, quizartinib inhibited the phosphorylation of ITD-FLT3 and its downstream molecules, STAT5, AKT and MAPK (Figure 3A). Although ITD-FLT3 was re-phosphorylated by the addition of FL, downstream molecules were not affected by FL. In contrast, FL activated Wt-FLT3, but not ITD-FLT3, in Wt- and ITD-FLT3 co-expressing cells in the presence of quizartinib. In paralleled with the activation of Wt-FLT3, AKT and MAPK, but not STAT5, were re-phosphorylated by FL (Figure 3A). In Wt- and cyITD-FLT3 co-expressing cells, FL also re-activated Wt-FLT3, AKT and MAPK, but not STAT5 (Figure 3A). These results indicated that FL activated Wt-FLT3, but not ITD-FLT3, in the co-expressing cells and confirmed again that the reduced inhibitory effects of FLT3 inhibitors were caused by FL-dependent Wt-FLT3 signals.

To examine a longer-time effect of FL on FLT3 inhibitors, we treated these cells with quizartinib and FL simultaneously for 2 hours. In Wt- and ITD-FLT3 co-expressing cells, the phosphorylation status of FLT3 and its downstream molecules was inhibited by quizartinib; however, the de-phosphorylation effects on AKT and MAPK, but not STAT5, were impaired in the presence of FL (Figure 3B). The same results were also obtained from Wt- and cyITD-FLT3 co-expressing cells (Figure 3B). In sole ITD-FLT3 cells, de-phosphorylation of FLT3 and its downstream molecules after treatment with quizartinib, were not affected by FL except for weak activation of AKT. These results indicated that reduced inhibitory effects by FL on Wt- and ITD-FLT3 co-expressing cells were mainly caused by the sustained activation of MAPK. On the other hand, in accordance with the results from the cell proliferation assay, de-phosphorylation effects of lestaurtinib in the presence of FL showed no significant difference on FLT3 kinase and its downstream molecules between sole ITD-FLT3 cells and Wt- and ITD-FLT3 co-expressing cells (Supplementary Figure 4).

To confirm the involvement of FL dependently activated Wt-FLT3 in the inhibitory effects on FLT3 inhibitors, we established kinase-dead (KD) FLT3 (K644A) and ITD-FLT3 co-expressing cells [22]. In KD- and ITD-FLT3 co-expressing cells, FL did not activate MAPK, nor affect growth inhibitory effect of quizartinib (Figure 3C-3D). These results collectively suggested that FL-dependent activation of Wt-FLT3 is a key mechanism in FL induced resistance of FLT3 inhibitors.

### FL reduces anti-leukemia effects of FLT3 inhibitors in primary AML cells

**Figure 4:**
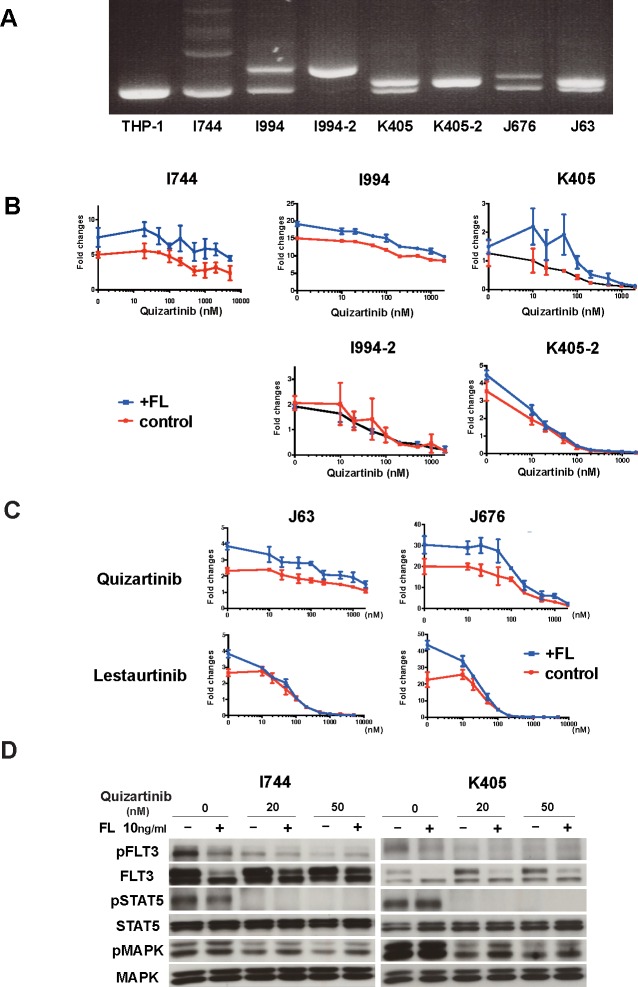
FL impaired the *in vivo* effects of FLT3 inhibitors and reduced the cytotoxic effects of FLT3 inhibitors in primary AML cells **A.** Detection of *FLT3*-ITD mutation in primary AML cells by PCR. THP-1 was served as a control for Wt-FLT3.**B.** Primary AML blast samples were incubated in MethoCult methylcellulose semisolid medium with increasing concentrations of the indicated FLT3 inhibitors for 7 days in the absence or presence of 50ng/ml FL. The data on day 7 are normalized to untreated controls on day 0. Different primary AML blast samples harboring both Wt-FLT3 and ITD-FLT3 (upper panels) and primary AML cells with a homozygous *FLT3*-ITD mutation (lower panels) were incubated with quizartinib. **C.** Inhibitory effect on AML blast samples harboring both Wt-FLT3 and ITD-FLT3 were compared between quizartinib and lestaurtinib. **D.** *FLT3*-ITD positive two AML patient samples were subjected to Western blot. Primary AML cells were incubated with the indicated concentration of quizartinib with or without FL for 2 hours and then the phosphorylation status of FLT3, STAT5, and MAPK was detected.

Since most AML cells with *FLT3* mutation co-express Wt-FLT3, especially at diagnosis, we further validated our findings in six primary AML cells. *FLT3*-ITD mutation was examined by PCR (Figure 4A). We examined the anti-leukemia effects of quizartinib with or without FL on primary AML cells by cell growth assay. In AML cells expressing both Wt- and ITD-FLT3, the addition of FL induced upward shifts in the dose-response curve, indicating increased cell viability and resistance to quizartinib (Figure 4B, upper panels). However, this FL-induced upward shift in the dose-response curve was not observed in the samples K405-2 and I994-2 with a homozygous ITD allele obtained after disease progression in a xenograft mouse model (Figure 4B, lower panels). In accordance with the results from 32D cells, FL-induced resistance was limited when AML cells expressing both Wt- and ITD-FLT3 were treated by lestaurtinib (Figure 4C). These results demonstrated that growth inhibitory effects of quizartinib were also reduced by FL in primary AML cells harboring a higher ratio of the Wt-FLT3 allele.

Next, we examined the effects of FL on downstream molecules from FLT3 in primary AML cells. We treated two primary AML cells with quizartinib in the presence of FL for 2 hours. In AML cells with high Wt-FLT3 ratios (I744 and K405), FL reduced the de-phosphorylation effects of quizartinib on MAPK, but not STAT5 (Figure 4D).

### Activation of the MAPK pathway plays a crucial role in FL-induced resistance in Wt- and ITD-FLT3 co-expressing cells

**Figure 5:**
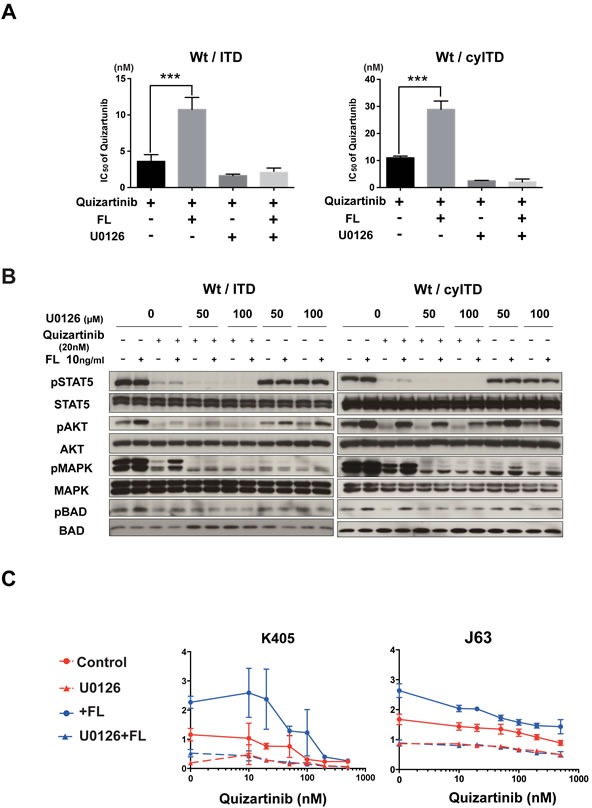
The FL-Wt-FLT3-MAPK axis is essential in reduced inhibitory effects of FLT3 inhibitors **A.** Wt- and ITD-FLT3 co-expressing 32D cells were treated with increasing concentrations of quizartinib and 40μmol/L (Wt- and ITD-FLT3 co-expressing cells) or 50μmol/L (Wt- and cyITD-FLT3 co-expressing cells) of U0126 with or without 50ng/ml FL for 48 hours, the GI_50_ values of quizartinib were calculated. **B.** Co-expressing 32D cells were treated with quizartinib and U0126 with or without FL for 2 hours, and then the phosphorylation status of STAT5, AKT, MAPK and BAD was detected. **C.** Two primary AML samples harboring both Wt- and ITD-FLT3 were subjected to evaluate the inhibitory effects of combination therapy with quizartinib and U0126 in the absence or presence of 50ng/ml of FL. The data on day 7 were normalized to untreated controls on day 0. Error bars represent the mean ± S.D. from four independent experiments (***P < 0.001).

Since our data showed that MAPK was the dominant pathway in the decreased inhibitory effect of FLT3 inhibitors, we investigated whether inhibition of MAPK by the MEK inhibitor U0126 canceled the FL-induced resistance to FLT3 inhibitors. As expected, addition of U0126 to quizartinib lowered GI_50_ values in sole ITD-FLT3 cells. Of note is that this combination nullified the increased GI_50_ value by FL stimulation in Wt- and ITD-FLT3 co-expressing cells (Figure 5A, left panel). We confirmed the same results in Wt- and cyITD co-expressing cells (Figure 5A, right panel).

We next investigated the effect of U0126 on activated MAPK pathways by FL stimulation. FL induced persistent phosphorylation of MAPK, as well as its downstream molecule BAD, in quizartinib treated Wt- and ITD-FLT3 co-expressing cells. On the other hand, cells treated with a combination of U0126 and quizartinib showed lower MAPK phosphorylation levels than quizartinib treated cells, and further activations of MAPK and BAD were not observed even after FL stimulation (Figure 5B). Furthermore, U0126 did not affect any other downstream molecules such as STAT5 and AKT, indicating that suppression of MAPK alone was sufficient to cancel the FL-induced resistance to quizartinib.

To determine the effect of U0126 on FL induced resistance to quizartinib in primary AML cells, we treated AML cells harboring both the ITD and Wt-FLT3 allele with quizartinib and U0126. MEK inhibition by U0126 cancelled the upward shift caused by FL in the dose-response curve in primary AML cells (Figure 5C).

### Surface expression of Wt-FLT3 is essential for FL induced resistance to FLT3 inhibitors

**Figure 6:**
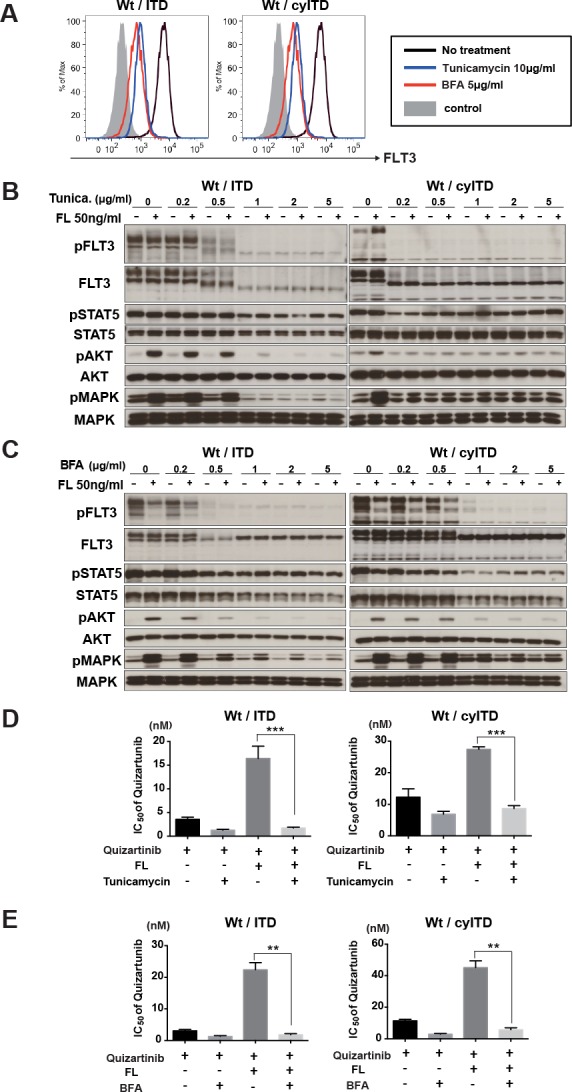
Cell surface localization of Wt- FLT3 is crucial to FL induced resistance to FLT3 inhibitors **A.** Transduced 32D cells, Wt- and ITD-FLT3 (left) and Wt- and cyITD-FLT3 (right) co-expressing cells were treated with Tunicamycin or BFA at the indicated concentrations for 16 hours. Then, cells were subjected to flow cytometer analysis for surface FLT3 expression. The graph shows representative results from 3 independent experiments. **B.** and **C.** Both Wt- and ITD-FLT3 co-expressing 32D cells were incubated with increasing concentrations of Tunicamycin **B.** or BFA **C.** for 16 hours, followed by FL stimulation for 10 minutes. Phosphorylation status of FLT3, STAT5, AKT, and MAPK were examined by Western blot. (D and E) Co-expressing 32D cells were incubated with various concentrations of quizartinib and 2μg/ml of Tunicamycin **D.** or BFA **E.** with or without 50ng/ml FL for 48 hours. The GI_50_ values of quizartinib were calculated. Error bars represent the mean values ± S.D. from at least three independent experiments (**P < 0.01, ***P < 0.001).

We demonstrated that FL stimulation to ITD-FLT3 did not further activate its downstream molecules and showed a relatively small impact on the inhibitory effect of FLT3 inhibitors. The localization of Wt- FLT3 and ITD-FLT3 enable us to explain this different response to FL stimulation. It was reported that the majority of ITD-FLT3 tends to exist in cytoplasm and activates its downstream molecules without ligand stimulation, while Wt-FLT3 requires binding of FL on the cell surface to activate itself and its downstream molecules [23]. It is known that maturation of FLT3 with glycosylation is necessary for cell surface expression. We examined whether blocking of FLT3 receptor maturation and translocation to the cell surface impeded FL-dependent activation of Wt-FLT3. Wt- and ITD-FLT3 co-expressing cells were treated with the glycosylation inhibitors tunicamycin or BFA. Flow cytometry analysis showed that both inhibitors reduced surface expression of FLT3 in Wt- and ITD-FLT3 co-expressing cells (Figure 6A); however, western blot analysis showed different glycosylation status of FLT3 between tunicamycin and BFA. Glycosylation status of FLT3 was completely blocked by tunicamycin, but it was partially by BFA (Figure 6B-6C). Both tunicamycin and BFA significantly blocked the FL induced activation of Wt-FLT3 and its downstream molecules, MAPK and AKT, although phosphorylation level of STAT5 was not impaired (Figure 6B-6C). Furthermore, FL-induced resistance to quizartinib was cancelled out by combination with tunicamycin or BFA in Wt- and ITD-FLT3 co-expressing cells and Wt- and cyITD-FLT3 co-expressing cells (Figure 6D-6E). These results indicated the expression of Wt-FLT3 on the cell surface was indispensable to FL-induced resistance to FLT3 inhibitors and reduced surface expression of Wt-FLT3 by glycosylation inhibitors attenuated the FL induced resistance to FLT3 inhibitors.

## DISCUSSION

We demonstrated that the FL-dependent Wt-FLT3 mediated signal is an essential mechanism in the reduced inhibitory effects of FLT3 inhibitors in clinical use. In addition, we showed that the continuous FL stimulation which was induced by the contact with FL-expressing stromal cells, also provides AML cells with resistance to FLT3 inhibitors in bone marrow. Since both soluble FL and membrane-bound FL stimulation are associated with the FL-dependent resistance to FLT3 inhibitors, plasma concentration of FL is not always parallel with this resistance. The stromal cells expressing FL also protects residual leukemia cells in bone marrow and gives rise to disease relapse. Therefore, the interaction between co-expressed Wt-FLT3 on AML cells and FL expressing stromal cells in bone marrow microenvironment should be considered for total eradication of *FLT3*-ITD positive AML cells.

Over the last decade, several FLT3 inhibitors, which possessed different characteristics, were investigated. Although all five FLT3 inhibitors evaluated in this study revealed elevated GI_50_ values in the presence of FL, the extent of resistance can be explained by selectivity, as well as potency, against Wt-FLT3 and ITD-FLT3. Tyrosine kinase inhibitors are classified into two types based on affinity patterns to receptors. Type I inhibitors bind to FLT3 both in active and inactive forms, whereas Type II inhibitors only bind to FLT3 in an inactive [24]. Type I inhibitors; midostaurin and Lestaurtinib tended to show the lesser impact of FL on their efficiency. Since these inhibitors have potency against multiple kinases, off-target effects might avoid the FL-dependent growth inhibitory effects. Furthermore, since quizartinib has a higher potency against ITD-FLT3 than Wt-FLT3, FL-dependent activation of Wt-FLT3 might reduce the inhibitory effect more apparently.

In *FLT3*-ITD positive AML, the ITD- to Wt-FLT3 allelic ratio varies among patients, and is thought to be associated with the sensitivity of FLT3 inhibitors. Pratz et al. reported that the mutant allelic ratio predicted the response to FLT3 inhibitors, and relapsed or refractory AML cells harbored a high mutant allelic burden [25]. Furthermore, it is thought that those relapsed cells tend to be more addictive to mutant FLT3 for cell survival. On the other hand, at diagnosis, most FLT3 mutated AML cells expressed Wt-FLT3 with a higher allelic burden of Wt-FLT3. This molecular difference between AML cells at diagnosis and relapse may explain the dismal efficacy of FLT3 inhibitors in newly diagnosed patients. Actually, we demonstrated that primary AML cells with higher wild-type allelic burden were much more affected by FL in resistance to FLT3 inhibitors *in vitro* and *in vivo*. Moreover, our findings also showed that an increased FL level facilitates resistance, especially in highly selective FLT3 inhibitors such as quizartinib and sorafenib.

In our observation, MAPK was the dominant pathway in the decreased inhibitory effects of FLT3 inhibitors induced by FL. Persistent MAPK activation leads to BAD phosphorylation at Ser^112^, resulting in the inhibition of cell apoptosis. It was also reported that growth factors widely induced resistance to primary kinase inhibitors by persistent activation of AKT and MAPK pathways in other cancers and this resistance can be overcome by secondary inhibitors targeting the receptors and its ligand. A study revealed that HGF activated the MET-ERK pathway, inducing resistance to erlotinib in an EGFR-mutated lung cancer cell line, KHM-3S, and the sensitivity of erlotinib was restored by crizotinib [26]. Straussman et al. reported that HGF derived from the tumor microenvironment elicit resistance to RAF inhibitors in BRAF-mutant melanoma through activation of MET signaling [27]. These studies also highlighted the importance of ligand-dependent Wt-RTKs activation in kinase inhibitor resistance.

Since FL has been considered one of the important mechanisms leading to lower clinical efficacies of FLT3 inhibitors, several strategies can be applied to restore the potency of FLT3 inhibitors. Our results suggested that FLT3 inhibitors with balanced potency against Wt-FLT3 and ITD-FLT3 or combination therapy with inhibitors to specific downstream molecules activated by FL can be a better therapeutic strategy. As an example, we indicated that inhibition of the MAPK pathway cancelled FL-induced resistance in the Wt- and ITD-FLT3 co-expressing cells, providing evidence for the potential combination therapy with FLT3 inhibitors and MEK/MAPK inhibitors [28].

We also propose a strategy to overcome FL-mediated resistance by blocking glycosylation. Our results showed that blocking of FLT3 receptor maturation and translocation to the cell surface by glycosylation inhibitors impede FL-dependent activation of Wt-FLT3 and resistance to FLT3 inhibitors in Wt- and ITD-FLT3 co-expressing cells. Importantly, activated STAT5 by ITD-FLT3 was not impaired by tunicamycin and BFA, inhibition of receptor maturation did not affected ITD-FLT3 signals. These results give us further evidence that Wt-FLT3 activation by FL on the cell surface is vital for FLT3 inhibitor resistance. Williams et al. reported that fluvastatin inhibited FLT3 glycosylation and blocked the activation of FLT3-ITD by FL [29]. Our results also supported the potency of blocking glycosylation therapy in FLT3 mutated AML. Therefore, this strategy is expected to provide an additive or synergistic effect with FLT3 inhibitors. Since AML cells with *FLT3*-ITD mutation co-express not only Wt-FLT3, but also other Wt-RTKs such as KIT and AXL [30, 31], inhibitory effects of FLT3 inhibitors might be impaired by their ligands.

In summary, we demonstrate that FL reduces the inhibitory effects of FLT3 inhibitors through the activation of Wt-FLT3 in *FLT3*-ITD mutated AML cells. We also indicate that the lower efficacy of FLT3 inhibitors in the clinical setting than that in experimental models might be caused by screening systems that focus on only the inhibitory effects against mutant-FLT3. Multikinase inhibitors have a risk for inducing adverse effects by their off-target potency, and the high potency against Wt-FLT3 causes severe bone marrow suppression. Therefore, it requires further investigations to validate the optimal dose and treatment schedule. Furthermore, it is also important to keep on developing novel agents. We believe that Wt- and mutant-FLT3 co-expressing cells are useful for developing an attractive therapeutic strategy with FLT3 inhibitors.

## MATERIALS AND METHODS

### Cell lines and cell culture

The human leukemia cell lines, MV4;11 was obtained from ATCC; MOLM14 was from Fujisaki Cell Center, Hayashibara Biochemical Laboratories (Okayama, Japan). The murine IL-3 dependent myeloid progenitor cell line, 32Dcl3 (hereinafter referred to as 32D) was obtained from the RIKEN Cell Bank (Tsukuba, Japan).

Human full-length Wt-FLT3 and ITD-FLT3 cDNAs were reported previously [6, 32, 33]. We introduced Myc- and FLAG-tag sequences at the C-terminus of Wt-FLT3 and ITD-FLT3 cDNAs, and also constructed extracellular domain-lacking ITD-FLT3 (cyITD-FLT3) cDNA with Myc-tag. Kinase-Dead (KD-) FLT3 (K644A) was generated using PrimeSTAR Mutagenesis Basal Kit (Takara Bio Inc., Kusatsu, Japan). These cDNAs were cloned into the pMX-IP vector (kindly provided by Professor Toshio Kitamura, Tokyo University, Japan), and transduced into 32D cells, as previously described [34]. We established six kinds of FLT3-expressing 32D cells: sole Wt-FLT3 expressing cells, ITD-FLT3-Myc and ITD-FLT3-FLAG co-expressing cells, Wt-FLT3-Myc and ITD-FLT3-FLAG co-expressing cells, Wt-FLT3-FLAG and cyITD-FLT3-Myc co-expressing cells, sole cyITD-Myc expressing cells and KD-FLT3-Myc and ITD-FLT3-FLAG co-expressing cells (Supplementary Figure 1). We verified that all of these established cells were transformed and proliferated equally in the absence of murine IL-3 (Supplementary Figure 5). Stable expression of each FLT3 protein in each cell line was confirmed by Western blotting using anti-Myc-Tag (9B11; Cell Signaling, Danvers, MA) and anti-FLAG (M2; Sigma-Aldrich, St Louis, MO) antibodies.

Human membrane-bound form FL cDNA was amplified by reverse transcriptase-mediated PCR. A purified fragment was cloned into the pMX-IP vector and transduced into COS-7 cells retrovirally. Transduced COS-7 cells were maintained in DMEM with 10% FCS and 4μg/mL puromycin.

### Antibodies, drugs and reagents

The polyclonal rabbit anti-FLT3 (C-20) antibody was purchased from Santa Cruz Biotechnology (Santa Cruz, CA). Anti-phospho-FLT3 (Tyr591), anti-phospho-STAT5 (Tyr694), anti-phospho-AKT (Ser473), anti-phospho-p44/42 MAPK (Thr202/Tyr204), anti-phospho-BAD (Ser112), anti-AKT, anti-MAPK, anti-BAD, anti-PIM1 and Sepharose Conjugated Myc-tag antibodies were purchased from Cell Signaling Technology (Beverly, MA). Anti-STAT5 antibody was from BD Transduction Laboratories (San Jose, CA). Anti-FLAG antibody (clone M2) and anti-FLAG M2 affinity gel were from Sigma-Aldrich (St.Louis, MO). Anti-mouse and anti-rabbit horseradish peroxidase antibodies were purchased from GE Healthcare (Buckinghamshire, UK).

Five FLT3 inhibitors were used in this study: quizartinib was purchased from MedChemExpress (Mommouth Junction, NJ), sorafenib was from Selleck Chemicals (Houston, TX), KW-2449 [33] was a generous gift from Kyowa Hakko Kirin Co., Ltd. (Tokyo, Japan), midostaurin (PKC412) was from Enzo Life Sciences (Plymouth Meeting, PA), lestaurtinib (CEP701) and U0126 were from Merck Millipore Corporation (Billerica, MA). Tunicamycin and brefeldin A (BFA) were purchased from Sigma-Aldrich. Recombinant human FLT3 ligand (FL) was purchased from R&D Systems, Inc.

### Patient samples

Informed consent was obtained from all patients according to the Declaration of Helsinki for banking and molecular analysis. Approval was also obtained from the ethical committees of Nagoya University. Bone marrow (BM) samples from patients with AML were subjected to Ficoll-hypaque density gradient centrifugation. Mutation of *FLT3* gene was examined as previously described [1].

### Growth inhibition assay

Cell viability was measured using the CellTiter96 Proliferation Assay (Promega, Madison, WI) according to the manufacturer's instructions. GI_50_ values were calculated using XLfit software (IDBS, Surrey, UK). Human primary AML cells were seeded in MethoCult H4534 methylcellulose semisolid medium containing cytokines (StemCell Technologies, Vancouver, Canada) with FLT3 inhibitors in the presence or absence of 50 ng/ml FL for seven days in triplicate, and cell viability was measured by the CelltiterGlo Luminescence Cell Viability Assay from Promega Corporation. For the co-culture assay, established 32D cells were co-cultured with COS-7 or FL-COS7 cells overnight in triplicate, followed by treatment with FLT3 inhibitors for the indicated time, and cell viability was determined by the CelltiterGlo Luminescence Cell Viability Assay.

### Apoptosis assay

Detection of apoptosis was performed using MEBCYTO-Apoptosis kit (MBL, Nagoya, Japan). Cells were prepared according to manufacturer's instruction and then analyzed using flow cytometer (FACSAria, BD Biosciences, San Jose, CA). For co-culture assay, cells were stained with APC-conjugated Annexin V and PI as reported [35].

### Immunoprecipitation and Immunoblotting

Cells were washed twice with cold PBS and suspended with CelLyticM (Sigma-Aldrich) containing protease and phosphatase inhibitors (Sigma-Aldrich). After incubation, lysates were centrifuged and the supernatants were mixed with 2×sample buffer. Proteins were separated by SDS-PAGE, and transferred to polyvinyl difluoride (PVDF) membranes (Millipore, Billerica, MA). Membranes were blocked with SuperBlock (TBS) blocking buffer (Thermo Fisher Scientific, Waltham, MA) and incubated with the indicated antibodies. After incubation with anti-mouse or anti-rabbit horseradish peroxidase antibodies, ECL Western Blotting Detection Reagents (GE Healthcare) was used to detect the signal.

For immunoprecipitation, supernatants were incubated with Myc-tag antibody (Sepharose Conjugate) or anti-FLAG M2 affinity gel. After centrifugation and washing with TBS-T, the protein-beads complexes were separated and subjected to immunoblotting as described above.

### Mouse xenograft transplantation model

NOD-SCID mice were purchased from CLEA Japan, Inc. (Tokyo, Japan) and kept under standard laboratory conditions according to the guidelines of the institute for laboratory Animal research, Nagoya University. This study was approved by the institutional ethics committee for laboratory animal use. A total of 1×10^7^ FLT3-mutant cells or Wt- and mutant FLT3 co-expressing cells were suspended in 100 μL of PBS and inoculated subcutaneously into a shaved area of 6 mice for each cell line. Seven days after inoculation, six mice from each cell line were randomly divided into vehicle and quizartinib treatment groups according to tumor volume. Quizartinib (1 mg/kg q.d.) was given orally for 13 days and tumor volume was measured every 3 days and calculated by the following formula: Tumor volume = D_L_×Ds×Ds×1/2 (D_L_, long diameter, Ds, short diameter).

### Statistical analysis

Data were statistically analyzed by a two-tailed students *t-*test using GraphPad Prism Ver.6 (GraphPad Software, Inc., La Jolla, CA), and differences with *P* values less than 0.05 were considered significant.

## SUPPLEMENTARY MATERIAL


