# Supplementary material for: Co-expression of wild-type FLT3 attenuates the inhibitory effect of FLT3 inhibitor on FLT3 mutated leukemia cells
Source: Oncotarget. 2016 Jun 17;7(30):47018–32. doi: 10.18632/oncotarget.10147 (PMC5216920; doi:10.18632/oncotarget.10147)
Supplement: Supplementary file 1 [file oncotarget-07-47018-s001.pdf]

Co-expression of wild-type FLT3 attenuates the inhibitory effect of FLT3 inhibitor on FLT3 mutated leukemia cells

Supplementary Material

Supplementary Figure 1

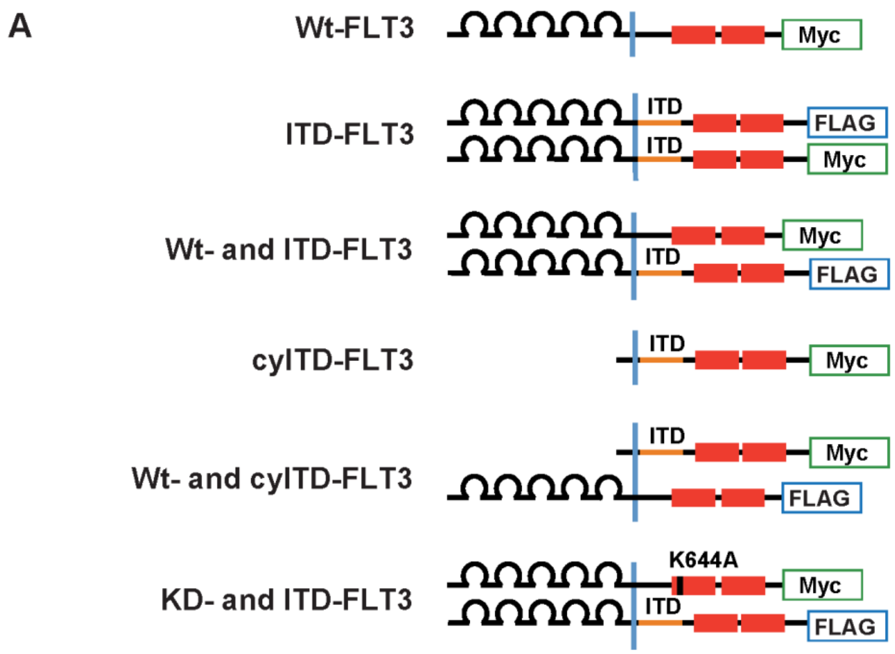

Supplementary Figure 1: Schema of established Wt- and/or ITD-FLT3 expressing 32D cells.

Supplementary Figure 2

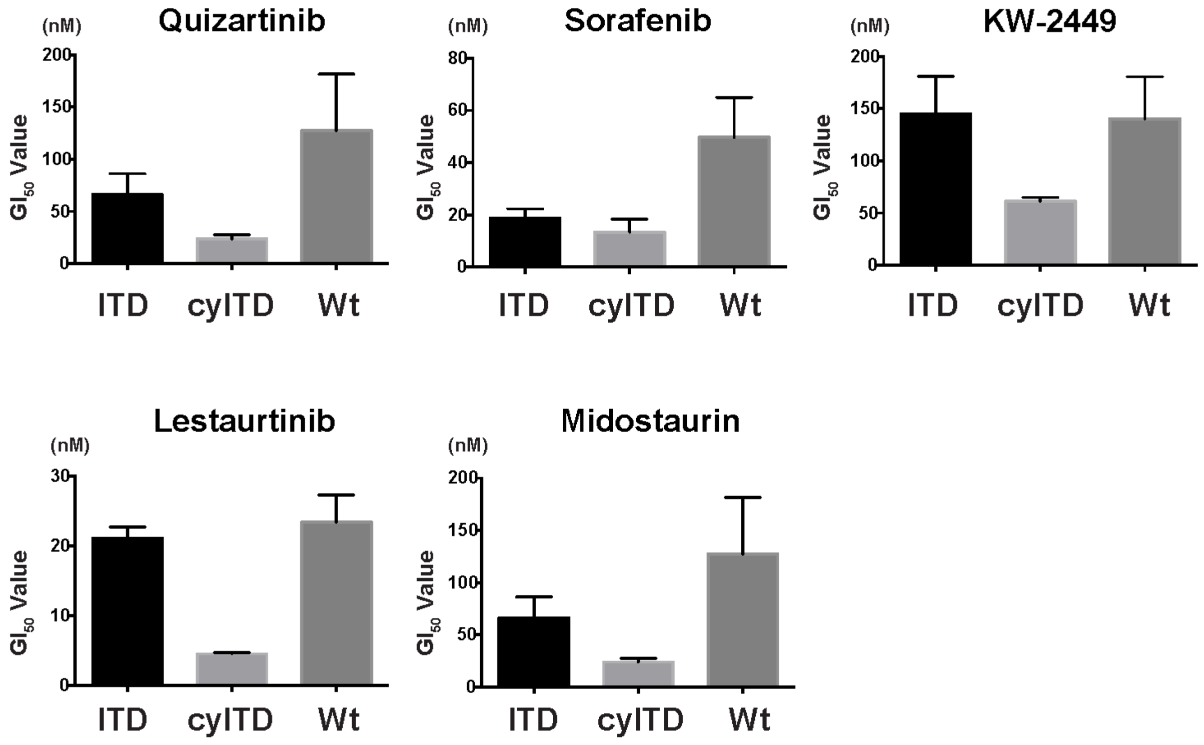

Supplementary Figure 2: GI50 values of five inhibitors against sole ITD-FLT3, sole cyITD-FLT3 and sole Wt-FLT3 expressing 32D cells were indicated.

Supplementary Figure 3

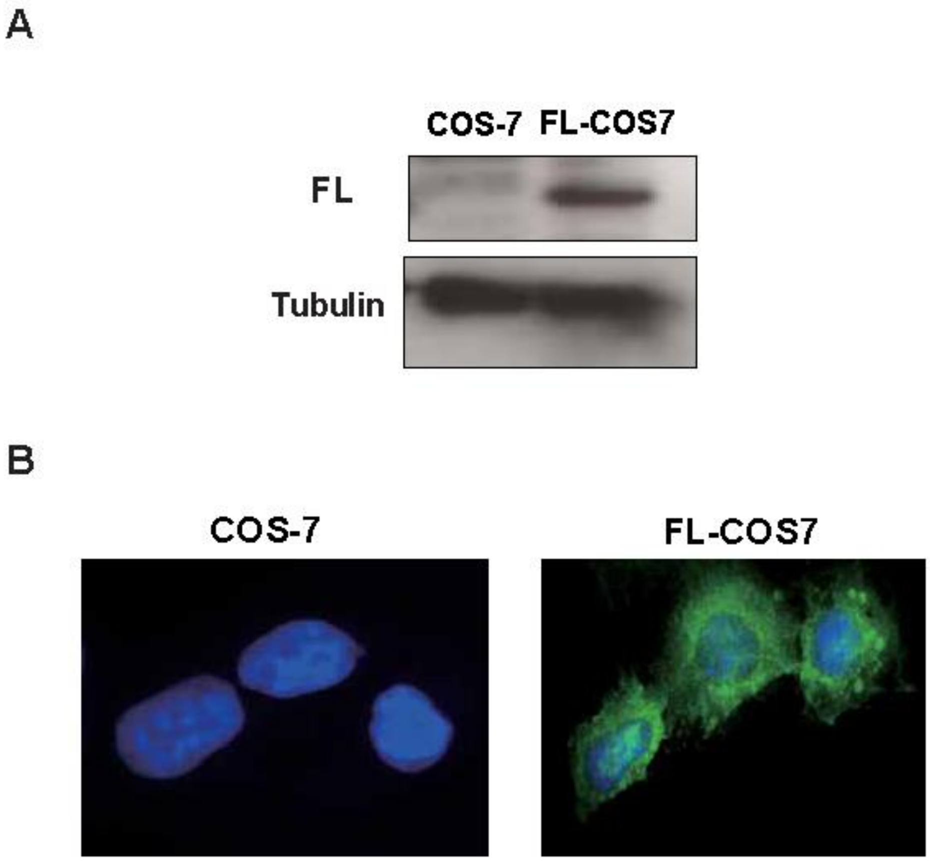

Supplementary Figure 3: Membrane-bound FL expression in established FL-COS-7 cells was examined.

(A) Western Blot **(B)** Immunofluorescence images

Supplementary Figure 4

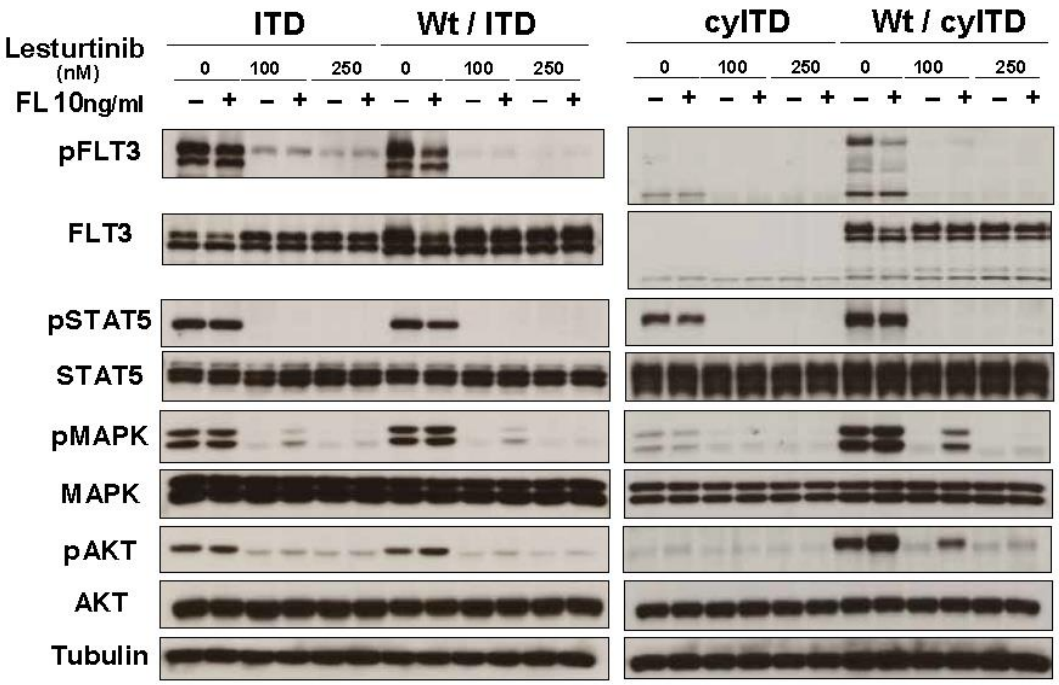

Supplementary Figure 4: Wt- and ITD-FLT3 co-expressing 32D cells were treated with lestaurtinib at the indicated concentrations with or without 10ng/ml FL for 2 hours. Phosphorylation levels of FLT3, STAT5, AKT and MAPK were detected by Western blot.

Supplementary Figure 5

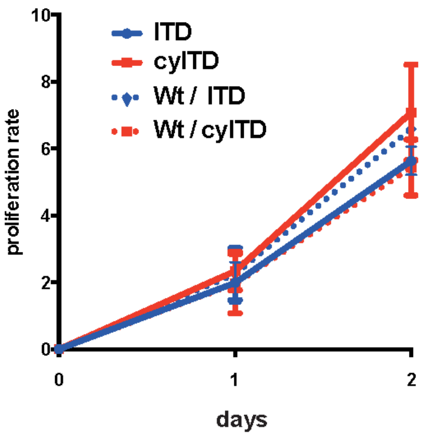

Supplementary Figure 5: Proliferation rate of established 32D cells in culture without murine IL-3.
